# Outdoor Cultivation of Marine Diatoms for Year-Round Production of Biofuels

**DOI:** 10.3390/md15040094

**Published:** 2017-03-25

**Authors:** Mitsufumi Matsumoto, Daisuke Nojima, Tomomi Nonoyama, Kiichi Ikeda, Yoshiaki Maeda, Tomoko Yoshino, Tsuyoshi Tanaka

**Affiliations:** 1 Biotechnology Laboratory, Electric Power Development Co., Ltd., 1, Yanagisaki -machi, Wakamatsu-ku, Kitakyusyu 808-0111, Japan; 2 Division of Biotechnology and Life Science, Institute of Engineering, Tokyo University of Agriculture and Technology, 2-24-16 Naka-cho, Koganei, Tokyo 184-8588, Japan; d-nojima@cc.tuat.ac.jp (D.N.); s161002q@st.go.tuat.ac.jp (T.N.); puchibou@hotmail.com (K.I.); y_maeda@cc.tuat.ac.jp (Y.M.); y-tomoko@cc.tuat.ac.jp (T.Y.)

**Keywords:** biofuel, diatoms, *Mayamaea* sp. JPCC CTDA0820, *F. solaris*, cold-tolerant microalgae, outdoor cultivation, open-pond bioreactor

## Abstract

Biofuel production using microalgae is believed to have the advantage of continuous year-round production over crop plants, which have strong seasonality. However, actual year-round production of microalgal lipids using outdoor mass cultivation has rarely been demonstrated. In our previous study, it was demonstrated that the oleaginous diatom, *Fistulifera solaris*, was culturable in outdoor bioreactors from spring to autumn, whereas biomass and lipid production in winter failed because *F. solaris* did not grow below 15 °C. Therefore, another candidate strain that is culturable in winter is required. In this study, a cold-tolerant diatom, *Mayamaea* sp. JPCC CTDA0820, was selected as a promising candidate for biofuel production in winter. Laboratory-scale characterization revealed that this diatom was culturable at temperatures as low as 10 °C. Subsequently, *F. solaris* (April–October) and *Mayamaea* sp. JPCC CTDA0820 (November–March) were cultured in outdoor open-pond bioreactors, wherein year-round production of diatom lipids was successfully demonstrated. The maximal values of areal productivities of biomass and lipids reached to 9.79 and 1.80 g/(m^2^ day) for *F. solaris*, and 8.62 and 0.92 g/(m^2^ day) for *Mayamaea* sp. JPCC CTDA0820, respectively. With the combined use of these two diatom species, stable year-round production of microalgal lipids became possible.

## 1. Introduction

Biofuel production from microalgae has attracted a great deal of attention because it offers a number of advantages over terrestrial crop plants [1]. It is estimated that microalgae exhibit higher lipid productivity than plants [2]. In addition, they do not compete with food and feed, thus enabling a stable supply of fuel resources [3]. In addition to these advantages, it is believed that microalgal biomass can be produced continuously year-round [4,5]. This useful feature could avoid seasonality, which reduces the biofuel production of terrestrial crop plants. However, although a number of studies have demonstrated outdoor cultivation of oleaginous microalgae [6,7,8,9,10,11,12,13,14], real-world year-round, outdoor cultivation of oleaginous microalgae has rarely been reported [12,13].

One of the critical factors in the success of year-round outdoor cultivation of microalgae is the temperature of the culture media [15]. Several studies [6,8,9,10] focused on outdoor cultivation in bioreactors with temperature control units. However, temperature control of a huge volume of culture media consumes a large amount of energy, which could degrade the energy balance of biofuel production; to minimize the energy input, it is preferable not to control the culture temperature [7]. For this reason, when we select the candidate strains for outdoor cultivation, the candidate strains should have not only high lipid content, but also robustness of cell growth over a wide range of temperatures. It has frequently been reported that some microalgae show steady growth even at high temperatures (approximately 40 °C) in outdoor cultivation [7,12,16]. By contrast, knowledge of the outdoor cultivation of microalgae at low temperatures is poorly consolidated. Although screening studies of cold-tolerant microalgae have recently been reported [17,18,19,20,21], to the best of our knowledge, the applicability of such microalgae for year-round outdoor cultivation has not been investigated.

Previously, we reported an outdoor cultivation study of the oleaginous diatom, *Fistulifera solaris* JPCC DA0580 [16]. This marine diatom accumulates a large amount of storage lipids in the form of triacylglycerol (TAG) [22]. Multiomics studies of this diatom have been conducted [22,23,24], and these investigations provided useful targets for genetic manipulation to further improve the efficiency of biofuel production [25,26]. The outdoor cultivation study conducted at Kitakyushu (33°92’73”N, 130°74’15”E) in Japan revealed that this diatom showed steady growth in raceway- and column-type bioreactors without temperature control from spring to autumn seasons (April–November). The temperature of the culture media reached 42 °C in the summer of the operation period; *F. solaris* was well adapted to such high-temperature environments. However, from December to March, the low temperatures inhibited cell growth. Therefore, year-round production of microalgal lipids solely by cultivating this diatom could not be demonstrated. To address this issue, we hypothesized that the combined use of *F. solaris* and another oleaginous marine diatom that are tolerant to low temperatures could be effective. By employing taxonomically close microalgae, it is expected that the two diatom species can share the same facilities for cultivation and subsequent down-stream processes such as cell harvesting, lipid extraction, and conversion. To test this hypothesis, we isolated 70 diatom strains—which were culturable at low temperatures—from a coastal area and a brackish lake in Japan, and identified a cold-tolerant and oleaginous marine diatom strain, namely, JPCC CTDA0820 [27].

In this study, we demonstrated that the diatom strain JPCC CTDA0820 belongs to the genus *Mayamaea*, based on molecular phylogenetic analysis. Subsequently, the cold-tolerant marine diatom, *Mayamaea* sp. JPCC CTDA0820 was investigated as a complementary lipid producer in the winter season. The growth characteristics of *Mayamaea* sp. JPCC CTDA0820 at low temperature were studied in laboratory-scale experiments, and compared to those of *F. solaris*. Outdoor mass cultivation of *Mayamaea* sp. JPCC CTDA0820 was then demonstrated in open-pond bioreactors (10–640 m^3^). Finally, year-round production of microalgal lipids was demonstrated by culturing *F. solaris* (April to November) and *Mayamaea* sp. JPCC CTDA0820 (December to March) in outdoor open-pond bioreactors. Environmental factors that affect the biomass and lipid productivities were discussed. This study provides useful information regarding the stable production of biofuels and outdoor mass cultivation of oleaginous diatoms.

## 2. Results

### 2.1. Molecular Phylogenetic Analysis of Mayamaea sp. JPCC CTDA0820

**Figure 1:**
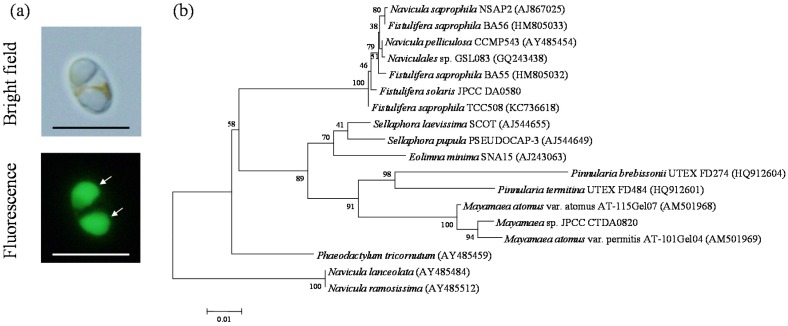
Microscopic images (**a**) and phylogenetic position (**b**) of JPCC CTDA0820. In the fluorescent microscopic images, oil bodies stained with BODIPY505/515 are indicated by arrows. Phylogenetic analysis was performed based on the 18S rDNA gene sequence data. Bootstrap values greater than 50 are shown on the nodes that were recovered in the maximum likelihood analysis.

We performed a screening study to obtain cold-tolerant diatoms that have an oleaginous phenotype [27]. Among the 70 diatom strains that were culturable in the half concentrated f-medium [28] (f/2 medium) at 10 °C, strain JPCC CTDA0820 showed the most obvious oleaginous phenotype, as confirmed by the BODIPY505/515 staining assay. This strain was isolated from the seawater obtained from a coastal area of Kitakyusyu, Fukuoka, Japan. The typical cells of *Mayamaea* sp. JPCC CTDA0820 were spheroid, and contained two oil bodies (Figure 1a). PCR amplification and subsequent DNA sequencing determined 1779 bp of the 18S rDNA gene for JPCC CTDA0820. Phylogenetic analysis indicates that three strains of *Mayamaea*, including JPCC CTDA0820, constituted a monophyletic group with a high bootstrap value of 91% (Figure 1b). This result suggests that JPCC CTDA0820 could belong to the genus *Mayamaea*. In this study, we describe this strain as *Mayamaea* sp. JPCC CTDA0820; however this designation is tentative.

### 2.2. Characterization of Mayamaea sp. JPCC CTDA0820 by Laboratory-Scale Indoor Cultivation

First, the growth of *Mayamaea* sp. JPCC CTDA0820 was characterized using microtiter plates (200 μL, under continuous illumination for 24 h, Figure S1). When we investigated the culture conditions of *Mayamaea* sp. JPCC CTDA0820 with different nutrient concentrations, better growth was observed in the modified f/2 medium with 4-fold silicate salts (N:P:Si mol-ratio = 24:1:12) as compared to the normal f/2 medium (N:P:Si mol-ratio = 24:1:3). *Mayamaea* sp. JPCC CTDA0820 grew well at a sea salt concentration of 0–7.4% and between pH value of 5–9, which overlaps with the culture conditions of *F.* *solaris* (salinity of 0.44%–3.5% and pH 7–9) (Figure S1) [29]. These results suggest that it is possible to culture *Mayamaea* sp. JPCC CTDA0820 and *F. solaris* in the same culture conditions. Hereafter, we used the f/2 media for *Mayamaea* sp. JPCC CTDA0820 and *F. solaris*, and the 4-fold strength of the silicate was supplied for *Mayamaea* sp. JPCC CTDA0820.

**Figure 2:**
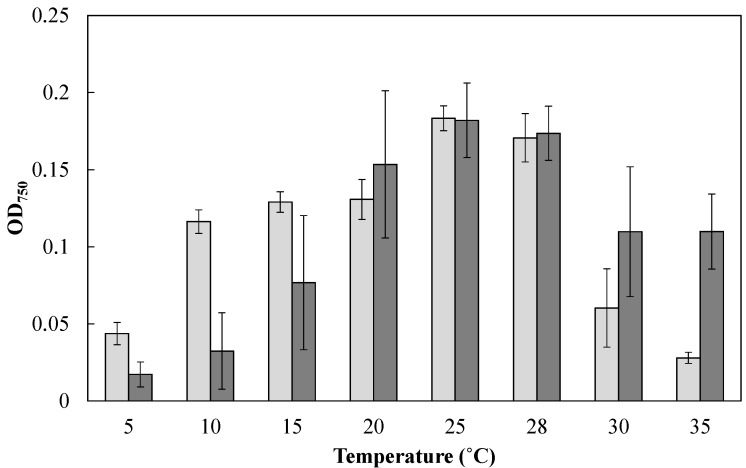
Effect of cultivation temperature on cell growth of *Mayamaea* sp. JPCC CTDA0820 (light gray) and *F. solaris* (dark gray). *Mayamaea* sp. JPCC CTDA0820 and *F. solaris* were cultured with modified-f/2 and f/2 media, respectively. The initial OD_750_ values were approximately 0.06, and decrease in OD_750_ was caused by cell death. The light intensity was 30 μE/(m^2^ s). Error bars represent standard deviations (*n* = 3).

To examine the effect of cultivation temperature on the cell growth of *Mayamaea* sp. JPCC CTDA0820 and *F. solaris*, these diatoms were cultured at various temperatures in the microtiter plates (200 μL, under continuous light for 24 h, Figure 2). The growth of *F. solaris* was maintained from 20 to 35 °C (the optimal temperature was 25 °C), but was inhibited below 15 °C, as has been previously reported [16,29]. In contrast, *Mayamaea* sp. JPCC CTDA0820 maintained its growth from 10 to 28 °C (the optimal temperature was 25 °C), and the growth was inhibited below 5 °C and above 30 °C.

Subsequently, biomass and lipid productivities of *Mayamaea* sp. JPCC CTDA0820 at 10 and 25 °C were investigated in indoor photobioreactors containing 500 mL of the modified f/2 medium. First, we tested whether a light-dark cycle affected the biomass and lipid productivities. In this experiment, CO_2_-enriched (2%) air was supplied. As a result, the volumetric productivities of biomass and lipids with a light-dark cycle (12 h:12 h of dark:light-140 μE/(m^2^ s)-cycle) were 293.7 ± 4.5 mg/L and 152.6 ± 23.3 mg/L (lipid content, 52.0 ± 1.4%) after 7 days of cultivation, whereas those under continuous light (24 h, 140 μE/(m^2^ s)) were 270.1 ± 91.5 mg/L and 127.1 ± 91.5 mg/L (lipid content of 47.1 ± 1.6%). Additionally, the impact of CO_2_ supply on the biomass and lipid productivities was examined by decreasing the CO_2_ concentration to 0.03% under a light-dark cycle. Results showed that the biomass and lipid productivities were 237 ± 87 mg/L and 77.1 ± 34.7 mg/L (lipid content of 31.2 ± 4.8%).

### 2.3. Year-Round Production of Diatom Lipids with Conbined Use of F. Solaris and Mayamaea sp. JPCC CTDA0820 in Open-Pond Bioreactors

**Figure 3:**
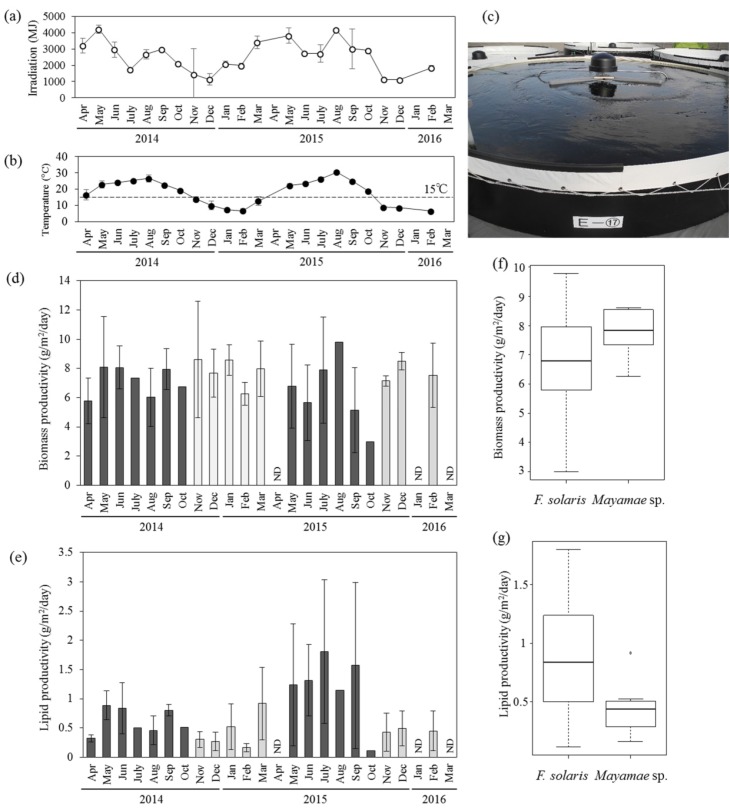
Outdoor cultivation of diatoms using 10 m^3^-scale open-pond bioreactors. (**a**) Variation of solar irradiation and (**b**) mean temperature of culture media. (**c**) Picture of the open-pond bioreactors. Variation of areal productivities of (**d**) biomass and (**e**) lipids of *F. solaris* (dark gray, April to October) and *Mayamaea* sp. JPCC CTDA0820 (light gray, November to March). ND means no data. Error bars represent standard deviations of each values obtain in each month. Monthly mean values of (**f**) biomass productivity and (**g**) lipid productivity of *F. solaris* and *Mayamaea* sp. JPCC CTDA0820 throughout the operation period are summarized in the boxplots.

Outdoor mass cultivation of *Mayamaea* sp. JPCC CTDA0820 and *F. solaris* was performed using open-pond bioreactors containing approximately 10 m^3^ of media (Figure 3, see also the Materials and Methods section for the bioreactor designs). Solar irradiation (Figure 3a) seasonally varied. The mean temperatures of the culture media (Figure 3b) in the 10 m^3^-scale open-pond bioreactor (Figure 3c) fell below 15 °C from November of 2014 to March of 2015, and from November of 2015 to February of 2016. The laboratory-scale experiments revealed that cell growth of *F. solaris* was inhibited at temperatures below 15 °C, whereas the cell growth of *Mayamaea* sp. JPCC CTDA0820 was not as low at 10 °C (Figure 2). Therefore, *Mayamaea* sp. JPCC CTDA0820 was cultured in the winter season (November of 2014 to March of 2015, November of 2015 to February of 2016), whereas *F. solaris* was cultured from spring to autumn (April to October of 2014 and May to October of 2015). Monthly mean values of areal productivities of biomass and lipid were within 3.00–9.79 g/(m^2^ day) and 0.11–1.80 g/(m^2^ day) for *F. solaris*, and 6.26–8.62 g/(m^2^ day) and 0.16–0.92 g/(m^2^ day) for *Mayamaea* sp. JPCC CTDA0820, respectively. These values can be converted to the volumetric productivities of 6.45–23.30 mg/(L day) and 0.25–3.42 mg/(L day) for *F. solaris*, and 12.45–17.47 mg/(L day) and 0.33–1.89 mg/(L^2^ day) for *Mayamaea* sp. JPCC CTDA0820, respectively. Overall, the biomass productivities of the two diatoms were comparable (Figure 3d,f), suggesting that year-round outdoor cultivation of diatoms was successfully achieved. *F. solaris* shows a higher level of lipid productivity than *Mayamaea* sp. JPCC CTDA0820 (Figure 3e,g). This phenomenon could be caused by a higher lipid content in *F. solaris* (2.8%–30.5%) than that in *Mayamaea* sp. JPCC CTDA0820 (2.6%–11.6%).

To determine the effect of the temperature of culture media and solar irradiation on the biomass productivity and lipid productivity of diatoms cultured outdoors, the correlations of these parameters were analyzed (Figure S2). When comparing the coefficients of correlation (Table S1), the biomass productivity, lipid productivity, and lipid content of *F. solaris* were more correlated with temperature than with solar irradiation. Indeed, lipid productivity of *F. solaris* was highly variable, even at the similar irradiation around 3000 MJ (Figure S2d). This is because these data points were obtained at different temperatures (ranging from 16.5 to 26.7 °C). In general, cultivation at relatively high temperatures yielded relatively high amount of lipids in this range. For *Mayamaea* sp. JPCC CTDA0820, biomass productivity was correlated with temperature, but was almost never correlated with solar irradiation. In contrast, lipid productivity and lipid content was strongly correlated with solar irradiation as compared to temperature. Factor loading plots based on principal component analysis (PCA) also indicate the same tendencies (Figure S3).

### 2.4. Comparison of Biomass and Lipid Production in Different Types of Outdoor Bioreactors

In our previous study [16], *F. solaris* was cultured outdoor in raceway- and vertical column-type bioreactors containing 200 L of culture media from April to November. Although simple comparison was difficult because of the different operation periods and culture volumes, *F. solaris* and *Mayamaea* sp. JPCC DA0820 in open-pond bioreactors (10 m^3^) produced biomass comparable to that of *F. solaris* in a vertical column (Figure S4). Areal lipid productivity from the open-pond was lower than that from the vertical column, but higher than that from the raceway (Figure S4). The open-pond bioreactor required less energy input to cultivate a unit weight of biomass than either the raceway- or column-type bioreactors (Figure S5).

### 2.5. Pilot-Scale Outdoor Cultivation of Mayamaea sp. JPCC CTDA0820

Pilot-scale outdoor cultivation of *Mayamaea* sp. JPCC CTDA0820 was performed by sequentially increasing the culture scale (40 m^3^, 160 m^3^, and 640 m^3^). We repeated this experiment twice: one experiment was performed from October 31 to November 21 of 2016 (Run 1), and the other experiment was performed from December 2 to 26 of 2016 (Run 2). The changes in temperatures and areal irradiations were shown in Figure S6. In Run 1, biomass production reached its maximum after 19 days of cultivation and subsequently decreased over time. Areal productions of biomass and lipids reached 89.75 g/m^2^ and 3.21 g/m^2^ after 14 days (i.e., the areal productivities of biomass and lipids were 6.41 g/(m^2^ day) and 0.23 g/(m^2^ day), respectively). Further cultivation resulted in decreases in biomass and lipid productivities to 64.86 g/m^2^ and 2.80 g/m^2^ after 21 days (i.e., the areal productivities of biomass and lipids were 3.09 g/(m^2^ day) and 0.13 g/(m^2^ day)). Over the same period, nitrate concentration decreased from 16.6 to 8.7 mg/L. Microscopic observation of the culture showed that the diatom cells had a characteristic brown color until day 14. Afterward, color-less cells increased in the culture over time. Additionally, contamination by amoeba-like microorganisms was observed. In Run 2, areal productions of biomass and lipids reached 75.77 g/m^2^ and 1.17 g/m^2^ after 17 days (i.e., the areal productivities of biomass and lipids were 4.46 g/(m^2^ day) and 0.07 g/(m^2^ day), respectively), and reached 83.10 g/m^2^ and 3.22 g/m^2^ after 24 days (i.e., the areal productivities of biomass and lipids were 3.46 g/(m^2^ day) and 0.13 g/(m^2^ day)). Over the same period, nitrate concentration decreased from 15.3 to 4.8 mg/L. The color-less cells were not observed during Run 2.

## 3. Discussion

In this study, *Mayamaea* sp. JPCC CTDA0820, which was obtained from a screening study of cold-tolerant oleaginous diatoms, was cultured with the goal of achieving year-round production of biofuels in combination with the mesophilic oleaginous diatom *F. solaris*. Characterization of cell growth of *Mayamaea* sp. JPCC CTDA0820 revealed that it is promising for stable cultivation at temperatures as low as 10 °C. This cell growth property can compensate for the disadvantages of the oleaginous diatom *F. solaris* in biofuel production, because *F. solaris* is not culturable below 15 °C, despite lower temperatures occurring during outdoor cultivation in winter [16]. Although the cold-tolerant character of the genus *Mayamaea* has not been comprehensively studied, it was previously reported that a closely related species, *Mayamaea atomus* var. permitis, showed tolerance to freezing at −20 °C [30], suggesting the potential of the genus *Mayamaea* for cultivation at low temperatures. Habitat environments can influence the tolerance to environmental stresses, including temperature change. *Mayamaea atomus* var. permitis, which exhibits tolerance to freezing, was isolated from moist, humus-rich soil [30], and thus, this strain is a terrestrial species that is believed to adapt to extreme terrestrial fluctuations in environmental conditions as compared to aquatic species [30,31]. By contrast, *Mayamaea* sp. JPCC CTDA0820 was isolated from a seawater sample obtained from a coastal area of Kitakyusyu, Fukuoka, where the average temperature of seawater rarely falls below 10°C. This observation is in good agreement with the cold-tolerance of *Mayamaea* sp. JPCC CTDA0820 that is demonstrated by laboratory-scale characterization.

**Figure 4:**
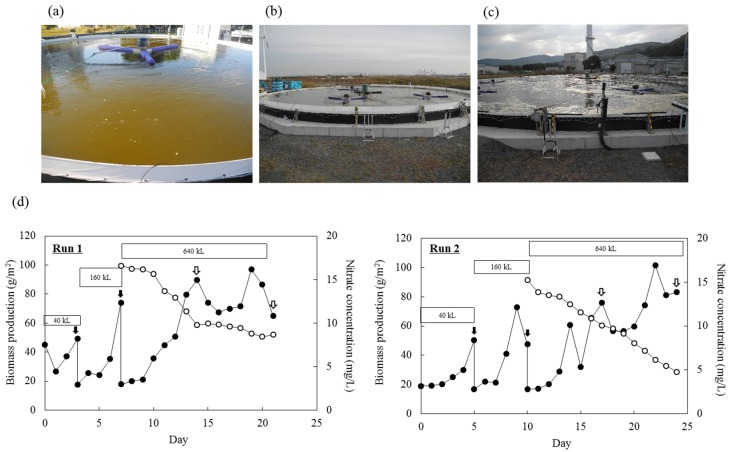
Pilot-scale outdoor cultivation of *Mayamaea* sp. JPCC CTDA0820S using the (**a**) 40 m^3^, (**b**) 160 m^3^, and (**c**) 640 m^3^-scale open-pond bioreactors. (**d**) Biomass (closed circles) was measured throughout the operation period. Cells were transferred to the bioreactors at larger-scale at the time indicated by the black arrows. Dry mass of the extracted lipids were measured at the time indicated by the white arrows. Nitrate concentration (open circles) was measured during cultivation in 640 m^3^ of culture media.

Subsequently, outdoor open-pond bioreactors (10 m^3^-scale) were operated to cultivate two diatoms—*F. solaris* and *Mayamaea* sp. JPCC CTDA0820—for approximately two years to achieve year-round production of biofuels. Culture scale was further expanded up to 640 m^3^ *Mayamaea* sp. JPCC CTDA0820. Areal productivities of biomass and lipids in 640 m^3^-scale open pond were comparable to those of 10 m^3^-scale open pond, indicating robust scalability of this system. Overgrowth of bacterial contamination, which might completely collapse the microalgal culture, was rarely observed despite the use of unsterilized culture media and open-pond reactors throughout the operation period. This result indicates that the growth rate of *F. solaris* and *Mayamaea* sp. JPCC CTDA0820 was high enough in seawater-based culture media, and that the growth of non-halophilic bacteria contaminating the outdoor bioreactor was suppressed, as discussed in our previous study [16]. By contrast, protozoal contamination was confirmed in the 640 m^3^-scale bioreactor once in the winter of 2016 (Figure 4), as previously observed when the outdoor raceway-type bioreactor was operated with *F. solaris* [16]. Therefore, future studies should focus on avoiding protozoal contamination [12].

*Mayamaea* sp. JPCC CTDA0820 showed lower values of areal lipid productivity than *F. solaris* (Figure 3f). Because the biomass productivity of these diatoms were comparable (Figure 3e), the lower lipid content of *Mayamaea* sp. JPCC CTDA0820 could have resulted in lower lipid productivity. In the present system, the lipid content of *Mayamaea* sp. JPCC CTDA0820 was strongly correlated with solar irradiation, and thus, light energy in winter season might not be sufficient to produce abundant lipids. It is well-known that photosynthesis can substantially affect the lipid synthesis by supplying the reducing equivalent (such as NADPH) for de novo synthesis of fatty acids. Improvement of light penetration might elevate the lipid productivity of *Mayamaea* sp. JPCC CTDA0820. Another possible way to improve the lipid content is to supply CO_2_ to the open-pond bioreactors. According to the laboratory scale characterization, a continuous supply of 2% CO_2_ resulted in a 1.24- and 2.06-fold increase in biomass and lipid productivity, as compared to the case with air supply (0.03% CO_2_). However, continuous CO_2_ supply requires electric power input throughout the bioreactor operation, which could negatively affect the energy production balance as discussed below.

Areal productivities of biomass and lipid for different types of outdoor bioreactors were also compared (Figures S4 and S5). Areal lipid productivity obtained from the vertical column-type bioreactor was higher than that from either the open-pond or raceway-type bioreactors (Figure S4). This result is reasonable for the following two reasons: (1) continuous air bubbling was supplied to the column-type bioreactor to agitate the cells, and (2) the footprint area of the vertical column was smaller than that of the raceway. However, there is one critical advantage of the open-pond bioreactor; namely, its low input electric power requirement for cultivation (Figure S5). Electric power was required primarily for the agitation of cells and CO_2_ supply. Agitation in the open-pond and raceway bioreactors was achieved by circulating propellers or waterwheels, whereas for the vertical column, agitation was performed by continuous air bubbling from the bottom of the vertical column. In the open-pond and raceway-type bioreactors, bubbling was only performed when pH needed to be adjusted by the CO_2_ supply. Therefore, the column-type bioreactor consumed the most electric power during cultivation. The open-pond and raceway-type bioreactors were operated using similar agitation and bubbling methods, whereas the raceway-type bioreactor showed lower biomass productivity (Figure S4). This could be caused by the bioreactor design of the open-pond with shallower depth, leading to shorter light path and thus more effective photosynthesis in individual cells. Therefore, we concluded that the open-pond bioreactor can produce biomass in the most effective manner among the bioreactors tested.

Areal productivities of biomass and lipid obtained in this study were compared to those of other oleaginous microalgae (Table 1). The biomass productivities in this study were comparable to those of other studies, whereas lipid productivity was relatively low. To further improve the lipid productivities, continuous CO_2_ supply is a promising strategy, as mentioned above, although it might reduce the advantage of low energy input. Therefore, we should consider methods that do not reduce this advantage, such as the use of CO_2_ generated from thermal power plants [32,33].

## 4. Materials and Methods

### 4.1. Isolation of Diatoms

Microalgae were isolated from seawater obtained from a coastal area of Kitakyusyu, Fukuoka, or ice samples collected from Lake Saroma, Hokkaido, Japan. The isolates were cultured in f/2 medium (mg): NaNO_3_ 75; NaH_2_PO_4_·2H_2_O 6; Na_2_SiO_3_·9H_2_O 40; ZnSO_4_·7H_2_O 0.021; MnCl_2_·4H_2_O 0.18; Na_2_MoO_4_·2H_2_O 0.0063; CoCl_2_·6H_2_O 0.01; CuSO_4_·5H_2_O 0.07; FeCl_3_·6H_2_O 3.16; Na_2_-EDTA 4.36; cyanocobalamin 0.0005; biotin 0.0005; thiamine·HCl 0.1; per 1 liter of artificial sea water at 4 °C. Subsequently, the cultures were spread on agar plates. The resulting colonies were cultured in f/2 medium at 10 °C, and BODIPY505/515 staining was conducted to detect intracellular lipid droplets. The stained diatom cells were washed and observed using fluorescent microscopy (BX51, Olympus Corporation, Tokyo, Japan) equipped with a cooled digital camera (DP-70, Olympus Corporation, Tokyo, Japan) to evaluate the oil accumulation [24,34].

### 4.2. Phylogenetic Analysis

A newly isolated strain was identified based on 18S rDNA sequences. The cells were incubated in deionized water at 100 °C for 5 min. The 18S rDNA was amplified by using a polymerase chain reaction (PCR) and the universal primer: forward primer, 5′-GGTGA TCCTG CCAGT AGTCA TATGC TTG-3′ and reverse primer, 5′-GATCC TTCCG CAGGT TCACC TACGG AAACC-3′. PCR reactions were carried out using PrimeSTAR Max DNA polymerase (TaKaRa Bio Inc., Shiga, Japan). Sequences of the 18S rDNA were determined by using the Applied Biosystems 3130xl Genetic Analyzer (Thermo Fisher scientific K.K., Kanagawa, Japan). The 18S rDNA sequence was compared to the 18S rDNA sequences derived from other diatoms by using the multiple sequence alignment program CLUSTAL W [35]. The maximum likelihood (ML) method was used to determine the 18S rDNA phylogeny. ML analysis was implemented by using MEGA5.2 software (Biodesign Institute, Tempe, AZ, USA) as previously reported [36]. Bootstrap trials were replicated 1000 times. Gaps were treated as missing data.

### 4.3. Laboratory-Scale Indoor Cultivation

First, *Mayamaea* sp. JPCC CTDA0820 was cultured in microtiter plates containing 200 μL of modified f/2 media with different nitrate, phosphate, and silicate concentrations, and different sea salt concentrations and pH at 10 °C for 5 days under continuous light for 24 h to investigate the optimal culture conditions. Then, *Mayamaea* sp. JPCC CTDA0820 and *F. solaris* were cultured in f/2 media, in which 4-fold strength silicate was supplied for *Mayamaea* sp. JPCC CTDA0820, at 5–35 °C in the microtiter plates (200 μL) under continuous light for 24 h to examine the impact of cultivation temperature on cell growth. Subsequently, *Mayamaea* sp. JPCC CTDA0820 was cultured in flat-flasks containing 500 mL of f/2 medium with 4-fold silicate with bubbling of CO_2_-enriched (2%) air at flow rate of 0.8 L/L/min under continuous light (140 μE/(m^2^ s), which can also be expressed as 140 μmol photon/(m^2^ s)) or light-dark cycle (12 h:12 h of dark:light-140 μE/(m^2^ s)-cycle) at 10 °C to examine the light conditions. Then, CO_2_ concentration decreased to 0.03% with the light-dark cycle.

### 4.4. Quantification and Statistics Analysis of Biomass and Oil Productivity

The cultured cells in 500 mL of medium were collected by centrifugation (8000 *g*, 10 min) and washed twice using deionized water. The cells were lyophilized (laboratory-scale) or heated at 100 °C (pilot scale) to measure their dry mass. The dried cells (approximately 50 mg) were disrupted using mortar and pestle. Disrupted cells were suspended in 8 mL *n*-hexane for oil extraction. The hexane fraction was recovered using centrifugation (1000 *g*, 10 min) and evaporated using an argon gas spray. Finally, the hexane extract material was determined by weighing. PCA was performed with statistical computing software, namely, R (ver3.2.5) (developed by the R Development Core Team, The R Foundation, Auckland, New Zealand).

### 4.5. Bioreactor Design and Culture Conditions for Outdoor Cultivation

Open pond-type bioreactors placed at Kitakyusyu, Fukuoka, Japan (Electric Power Development Co., Ltd., Fukuoka, Japan) were used for outdoor cultivation (Figure 3c and Figure 4a–c). Four types of bioreactors with different sizes (approximately 5, 10, 20, and 40 m in diameter) were used to cultivate the diatoms with approximately 10, 40, 160, and 640 m^3^ of culture, in which the initial water depth was approximately 0.5 m. Natural seawater were used to prepare the culture media for 10 to 40 m-scale open-pond bioreactors. Temperature and pH of the culture media were logged, and CO_2_ gas was supplied to keep the pH value at 9.0. The solar irradiance was measured using a general illuminance meter placed in the same site of the open pond bioreactors. Agitation of the culture media was performed by circulating screws (one for the 5- and 10-m ponds, four for the 20-m pond, and nine for the 40-m pond).

## 5. Conclusions

*Mayamaea* sp. JPCC CTDA0820 was identified as a promising candidate for biofuel production at low temperatures. Indoor cultivation tests revealed that this diatom was culturable at as low as 10 °C, at which another oleaginous diatom, *F. solaris*, cannot grow. Subsequently, year-round production of microalgal lipids was successfully demonstrated by cultivating two species of diatoms, *F. solaris* (April to October) and *Mayamaea* sp. JPCC CTDA0820 (November to March), in outdoor open-pond bioreactors. The cultivation scale ranges from 10–640 m^3^. Note that the open-pond bioreactor could produce microalgal biomass with less electric energy input than raceway- and column-type bioreactors which were previously used for mass cultivation of *F. solaris*. The biomass productivity obtained in this study was comparable to that of previous studies, whereas lipid productivity was relatively low. To increase the lipid content, methods involving the addition of CO_2_ are promising.

## Figures and Tables

**Table 1:** Comparison of areal productivities of biomass and lipids of microalgae cultured in outdoor bioreactors.

Microalgae	Outdoor Bioreactors	Biomass Productivity [g/(m^2^ day)]	Lipid Productivity [g/(m^2^ day)]	Reference
*Mayamaea* sp. JPCC CTDA0820	Open-pond	8.62	0.92	This study
*Fistulifera solaris*	Open-pond	9.79	1.80	This study
*Chlorella sorokiniana*	Column	10 ± 2.2	-	[7]
*Nannochloropsis* sp. F&M-M24	Green Wall Panels	9.9	6.5	[14]
*Tetraselmis suecica* F&M-M33	Green Wall Panels	7.6	1.7	[14]
*Tetraselmis* sp. MUR-233	Open-pond	7.2 ± 0.4	3.0 ± 0.6	[11]
*Tetraselmis* sp. MUR-233	Coiled tube	7.8 ± 0.2	5.0±0.4	[11]
*Pleurochrysis carterae*	Raceway	3.20–33.68	1.54–11.41	[12]
Microalgae in wastewater treatment high rate algal ponds		5.9	1.42	[13]

## Supplementary Materials

The following are available online at www.mdpi.com/1660-3397/15/4/94/s1, Figure S1: Culture conditions of *Mayamaea* sp. JPCC CTDA0820. Figure S2: Areal productivities of biomass and lipids of *F. solaris* and *Mayamaea* sp. JPCC CTDA0820 versus average culture temperature and solar irradiation. Figure S3: Factor loadings plots based on principal component analysis (PCA) with the parameters of biomass productivity, lipid productivity, lipid content, monthly mean temperature of the culture medium, and solar irradiation. Figure S4: Comparison of monthly mean values of (a) biomass productivity and (b) lipid productivity of *F. solaris* and *Mayamaea* sp. JPCC CTDA0820 cultured in the open pond bioreactor, raceway-type bioreactor, and column-type bioreactor. Figure S5: Comparison of monthly mean values of electric power required to cultivate a unit weight of biomass of *F. solaris* and *Mayamaea* sp. JPCC CTDA0820 cultured in open pond bioreactor (this study), raceway-type bioreactor, and column-type bioreactor. Table S1: Coefficient of correlation of biomass productivity, lipid productivity, and lipid content of *F. solaris* and *Mayamaea* sp. JPCC CTDA0820 cultivated outdoors using open-pond bioreactors (10 m^3^) versus average culture temperature and solar irradiation.
