# Supplementary material for: Outdoor Cultivation of Marine Diatoms for Year-Round Production of Biofuels
Source: Mar Drugs. 2017 Mar 25;15(4):94. doi: 10.3390/md15040094 (PMC5408240; doi:10.3390/md15040094)

Supplementary Information

Figure S1. Culture conditions of *Mayamaea* sp. JPCC CTDA0820. The diatom cells (initial cell concentration of 1.0 × 10^6 cells/mL, initial OD_{750} of approximately 0.06) were cultured at 10 °C for 5 days in 200 μL of culture medium with different (a) nitrate concentration, (b) phosphate concentration, (c) silicate concentration, (d) sea salt concentration, and (e) pH. Subsequently, optical densities (OD_{750}) were measured. Unless otherwise stated, growth conditions were as follows: nitrate concentration, 0.88 mM; phosphate concentration, 0.04 mM; silicate concentration, 011 mM; sea salt concentration, 3.7%; and pH8, which are same for the normal f/2 medium (indicated by underbars for each conditions). The light intensity was 30 μE/(m^2·s). Decrease in OD_{750} was caused by cell death. Error bars represent standard deviation (n = 3).

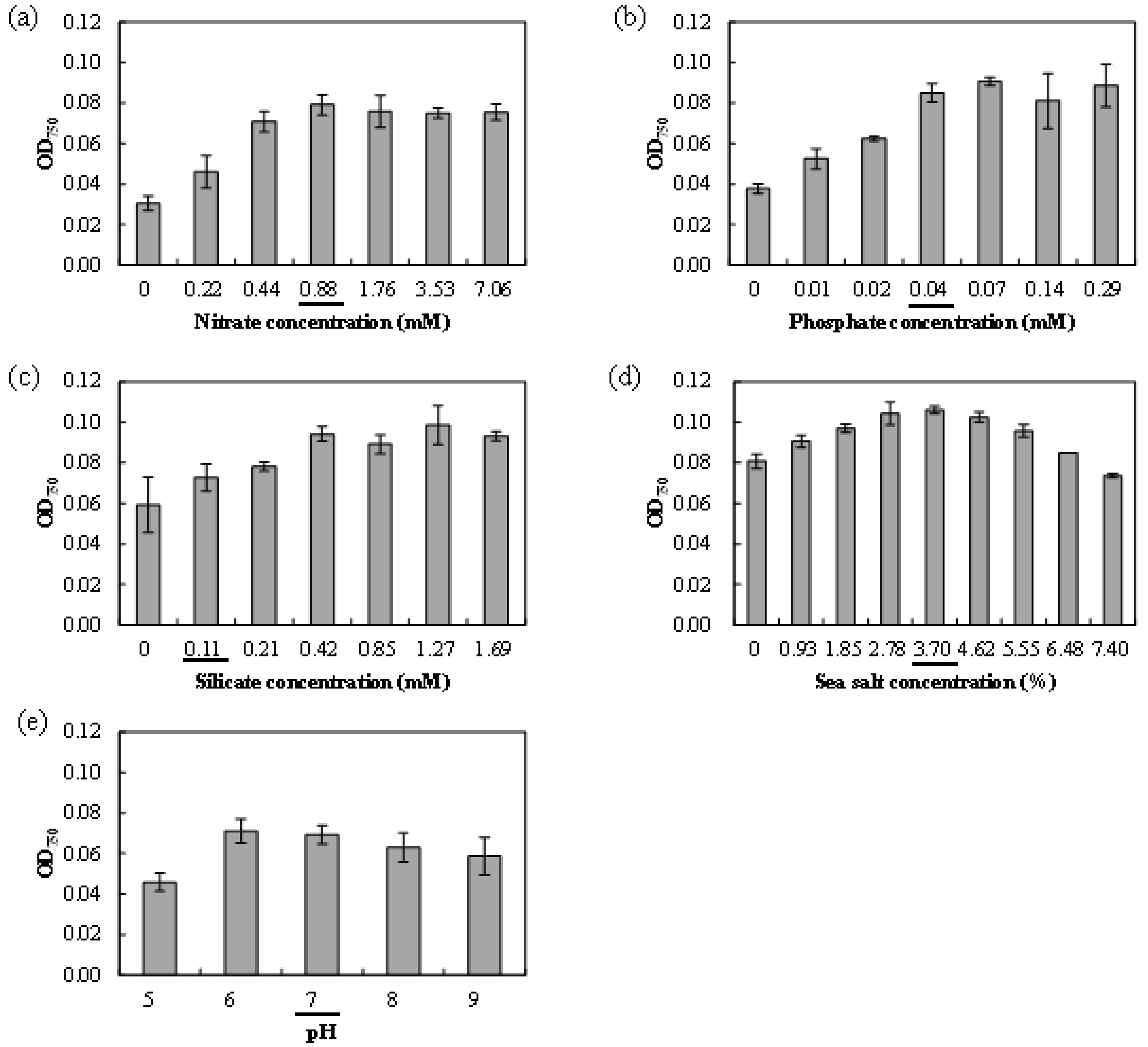

Figure S2. Areal productivities of biomass and lipids of *F. solaris* and *Mayamaea* sp. JPCC CTDA0820 versus average culture temperature and solar irradiation. Scatter plots show the correlations of biomass productivity and lipid productivity of *F. solaris* (closed circles) and *Mayamaea* sp. JPCC CTDA0820 (open circles) cultivated outdoors using open-pond bioreactors containing 10 m^3 media versus average culture temperature and solar irradiation.

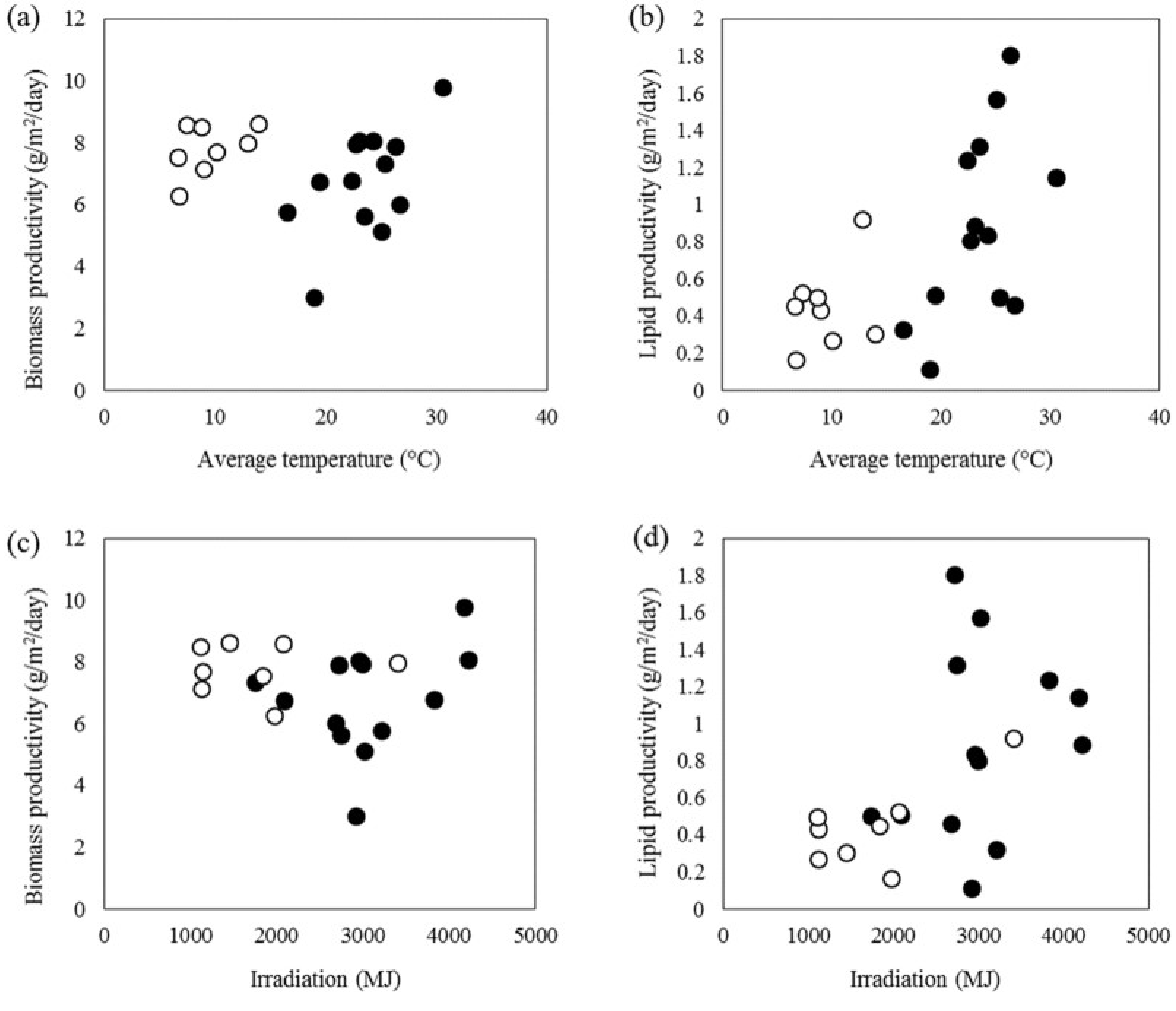

Table S1. Coefficient of correlation of biomass productivity, lipid productivity, and lipid content of *F. solaris* and *Mayamaea* sp. JPCC CTDA0820 cultivated outdoors using open-pond bioreactors containing 10 kL media versus average culture temperature and solar irradiation.

Production season (Species)	Productivity	Environmental conditions	
		Cultivation temperature (°C)	Solar irradiation (MJ)
Year-round (*Fistulifera solaris and Mayamaea* sp JPCC CTDA0820)	Biomass productivity	-0.08	-0.05
	Lipid productivity	0.61	0.53
	Lipid content	0.57	0.46
Spring-Autumn (*Fistulifera solaris*)	Biomass productivity	0.59	0.32
	Lipid productivity	0.54	0.26
	Lipid content	0.36	0.13
Winter (*Mayamaea* sp. JPCC CTDA0820)	Biomass productivity	0.47	-0.01
	Lipid productivity	0.32	0.71
	Lipid content	0.29	0.73

Figure S3. Factor loadings plots based on principal component analysis (PCA) with the parameters of biomass productivity, lipid productivity, lipid content, monthly mean temperature of culture medium, and solar irradiation. (a) Data obtained from year-round experiment with both *F. solaris* and *Mayamaea* sp. JPCC CTDA0820 used for PCA. (b) Data obtained from spring to autumn (with *F. solaris*) used for PCA. (c) Data obtained in winter (with *Mayamaea* sp. JPCC CTDA0820) used for PCA.

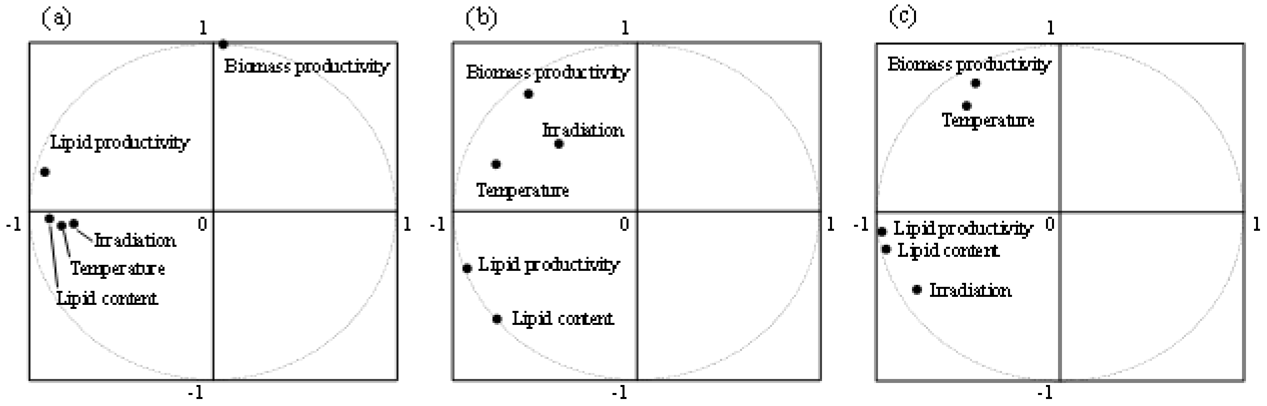

Figure S4. Comparison of monthly mean values of (a) biomass productivity and (b) lipid productivity of *F. solaris* and *Mayamaea* sp. JPCC CTDA0820 cultured in the open pond bioreactor (this study), raceway-type bioreactor, and column-type bioreactor. The data for the raceway-type bioreactor and column-type bioreactor were obtained from our previous study [15]. Boxplots were generated by R (ver. 3.2.5). The while dots represent the outliers which are defied as the values more than 1.5 interquartile ranges below (IQR shown by boxes) the first quartile or above the third quartile. The end of whiskers represent the minimal and maximal values among the analyzed values except for outliers. The bold lines in the boxes represent medians.

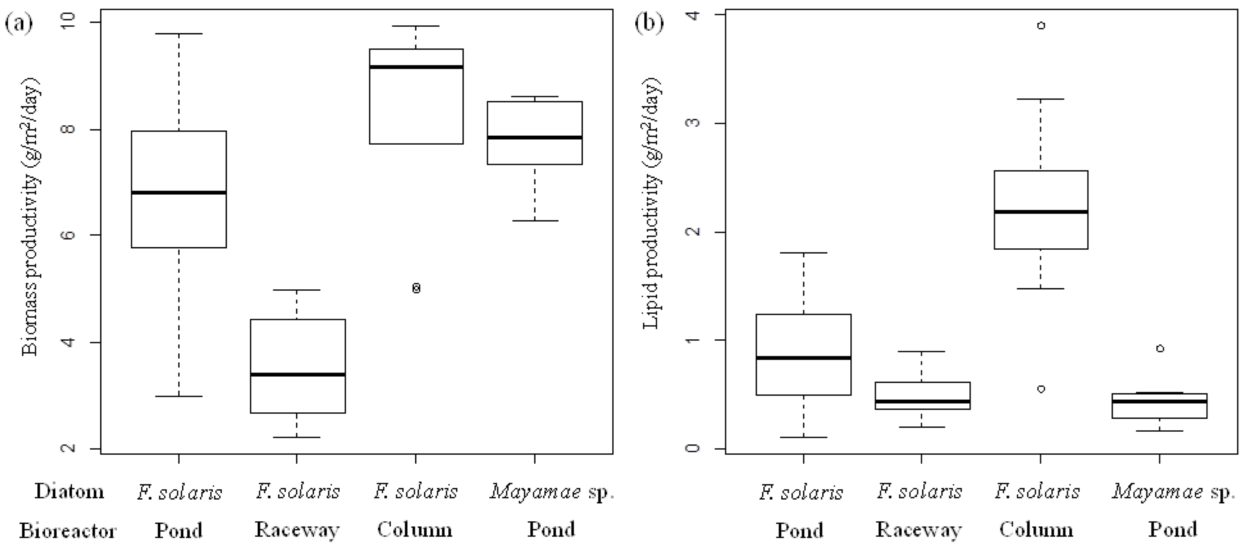

Figure S5. Comparison of monthly mean values of electric power supply required to cultivate a unit weight of biomass of *F. solaris* and *Mayamaea* sp. JPCC CTDA0820 cultured in open pond bioreactor (this study), raceway-type bioreactor, and column-type bioreactor. The data for the raceway-type bioreactor and column-type bioreactor were obtained from our previous study [15]. Boxplots were generated by R (ver. 3.2.5). The while dots represent the outliers which are defied as the values more than 1.5 interquartile ranges below (IQR shown by boxes) the first quartile or above the third quartile. The end of whiskers represent the minimal and maximal values among the analyzed values except for outliers. The bold lines in the boxes represent medians.

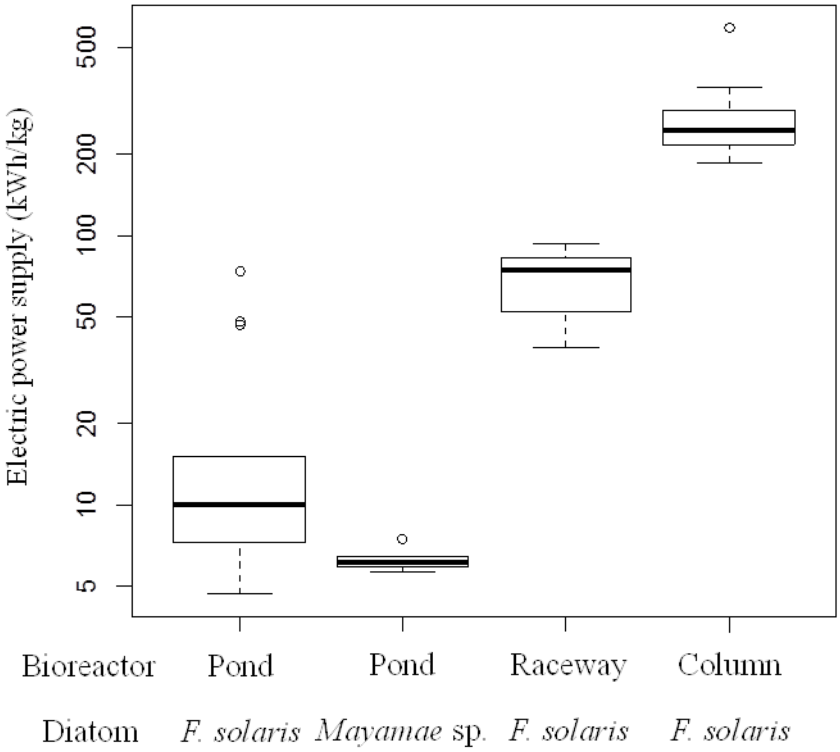

Figure S6. Changes in temperatures and areal irradiations during the 2 runs of pilot-scale outdoor cultivation of *Mayamaea* sp. JPCC CTDA0820

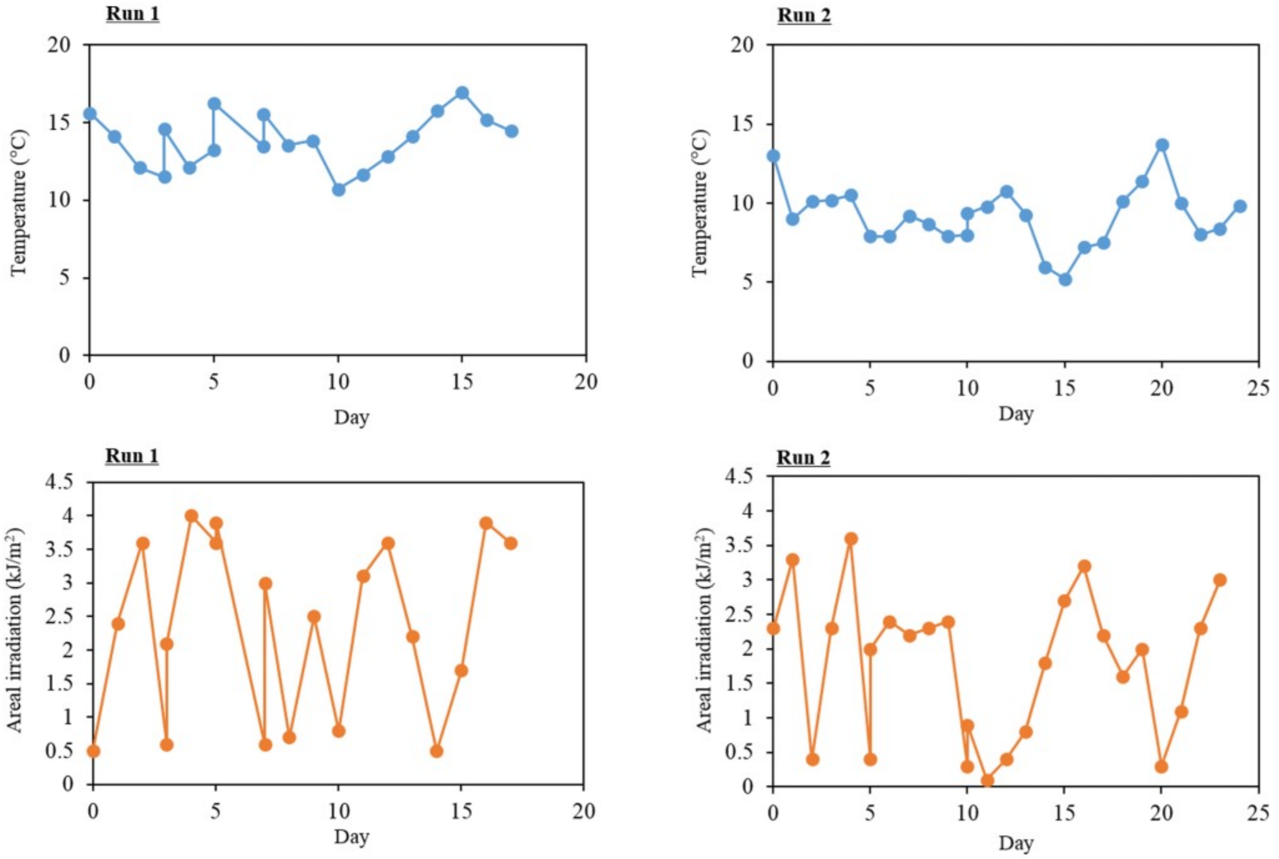

Supplement: Supplementary file 1 [file marinedrugs-15-00094-s001.pdf]
